# Multifocal glioblastoma—two case reports and literature review

**DOI:** 10.1186/s41016-020-00223-z

**Published:** 2021-01-15

**Authors:** Zuo-Xin Zhang, Ju-Xiang Chen, Bao-Zhong Shi, Guang-Hui Li, Yao Li, Yan Xiang, Xun Qin, Lin Yang, Sheng-Qing Lv

**Affiliations:** 1 Department of Neurosurgery, Xinqiao Hospital, Third Military Medical University, No.183 Xinqiao Street, Shapingba District, Chongqing City, 400037 People’s Republic of China; 2 Department of Neurosurgery, Changzheng Hospital and Shanghai Institute of Neurosurgery, Second Military Medical University, Shanghai, 200003 People’s Republic of China; 3 Department of Critical Care Medicine & Department of Neurosurgery, The First Affiliated Hospital, College of Clinical Medicine of Henan University of Science and Technology, Luoyang, 471003 Henan People’s Republic of China; 4 Institute for Cancer Research in People’s Liberation Army, Xinqiao Hospital, Third Military Medical University, Chongqing, 400037 People’s Republic of China

**Keywords:** Multifocal glioblastoma, Surgical resection, Radio-chemotherapy

## Abstract

**Background:**

Multifocal glioblastoma is a rare type of glioblastoma with worse prognosis. In this article, we aimed to report two cases of classical multifocal glioblastoma.

**Case presentation:**

In case 1, a 47-year-old male presented with dizziness, and once had a sudden loss of consciousness accompanied by convulsion of limbs. Contrast-enhanced MRI showed multiple lesions with heterogeneously ring-enhanced characters in the left hemisphere, diagnosed as multifocal glioblastoma. He underwent a craniotomy of all lesions, concurrent radiotherapy and chemotherapy as well as additional chemotherapy of temozolomide. After 2 cycles, repeat MRI showed that the new lesions already occurred and progressed. Eventually, he abandoned the chemotherapy after the 2 cycles and died 1 year later. In case 2, a 71-year-old male presented with a history of headache, left limb weakness, and numbness. Discontinuous convulsion of limbs once occurred. Contrast-enhanced MRI showed multiple lesions located in the right hemisphere, diagnosed as multifocal glioblastoma. He underwent a right frontoparietal craniotomy of the main lesion. Hemorrhage of the residual tumor and pulmonary artery embolism occurred synchronously. Eventually, his family decided not to pursue any further treatment and opted for hospice care and he passed away within 11 days of surgery.

**Conclusions:**

We reported two cases of typical multifocal glioblastoma. Valid diagnosis is crucial; then, resection of multiple lesions and canonical radio-chemotherapy probably bring survival benefits.

## Background

Glioblastoma multiforme (GBM) is the most common primary malignancy in central nervous system with high aggressiveness and extremely poor prognosis [1]. Although surgical resection with maximum safe range and additional radio-chemotherapy remains the standard treatment of GBM, the median survival of GBM is approximately less than 15 months [2]. Nowadays, the brain metastases, such as lung cancer, which is the most common source [3], are becoming more prevalent and common than primary brain cancer [4]. And the characteristic radiologic imaging feature of brain metastases, which is similar with the other diseases of intracranial multiple lesions, always results in misdiagnosis in the clinical management.

Multifocal glioblastoma is a rare type of glioblastoma multiforme and it associates with worse prognosis compared with solitary ones [5]. It manifests as multiple distinct lesions simultaneously, exhibiting a clear pathway of spread lesions [6] and there is a presumed microscopic connection among them [7]. It possesses the similar properties of radiologic imaging and can be sometimes misdiagnosed as brain metastases, which creates enormous challenges in the subsequent management.

Here, we present two cases of typical multifocal glioblastoma and we also have reviewed related literatures, and the aim of this report is to discuss the diagnosis and treatment of this peculiar malignancy.

## Case presentation

### Case 1

A 47-year-old male presented with dizziness for 10 days, and he also complained that he once had a sudden loss of consciousness accompanied by convulsion of his limbs which lasted for 3 min at the this duration. Syndromes of headache, vomiting, and hypoplasia were not described. Mild cognitive impairment and weakness of the right side were detected at admission.

**Fig. 1:**
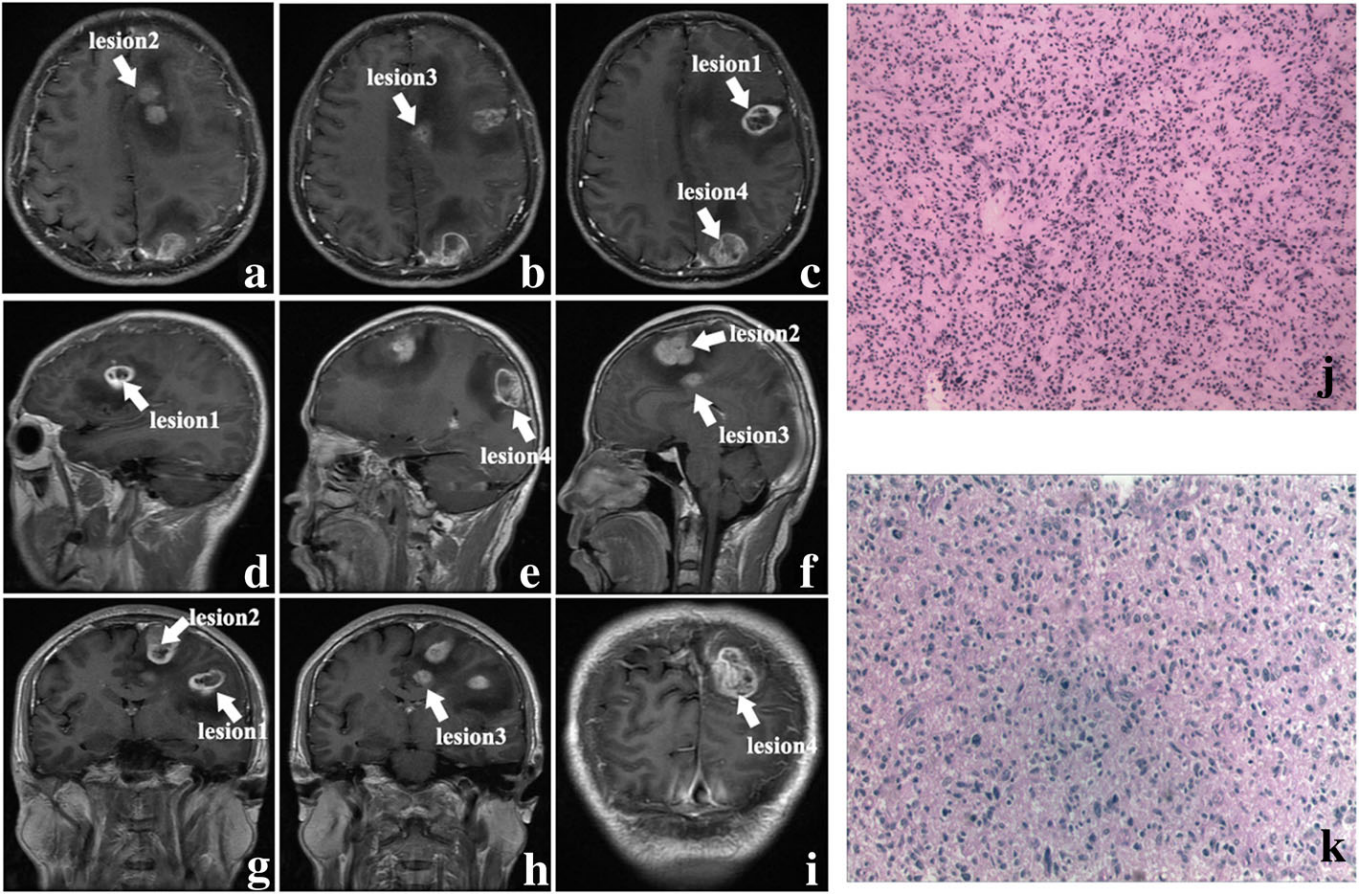
The axial, sagittal, and coronal contrast-enhanced MRI images (**a**–**i**) (arrows represent the lesions). The hematoxylin-eosin (HE) staining of intraoperative frozen histologic section and paraffin section (**j**, **k**, × 100)

Computed tomography (CT) of the brain were performed, which manifested multiple intracranial lesions synchronously. Positron emission tomography-computed tomography (PET-CT) revealed no abnormal neoplasms in the other tissues of the body. The magnetic resonance imaging (MRI) revealed 4 occupying lesions with surrounding edema in the left frontal and parietal lobes. Deep-seated lesions invaded to the parenchyma adjacent to the left ventricle, contributing to the mass effect and middle line shift. The whole masses presented with a heterogeneously ring-enhanced character with necrosis and cystic changes after gadolinium (Gd) administration (Fig. 1**a**–**i**).

The patient underwent a left frontoparietal craniotomy of 3 lesions with the guidance of intraoperative navigation and sodium fluorescence-guided technique. Lesions were seen with grey appearance, obscure boundary, and rich blood supply and removed for decompression of the intracranial pressure. We successfully resected the masses micro-surgically for additional biopsy. Then, the gross total resection of these masses around their approximate boundary was achieved. We used the same way to resect the last lesion in occipital lobe under prone position.

Histopathological examination revealed that these tumor cells possessed increased necrosis, and significant heterogeneity of nucleus (Fig. 1**j**, **k**). Immunohistochemical staining demonstrated that these lesions shared the similar characters of intensity of the biomarkers such as glial fibrillary acidic protein (GFAP) and Olig-2. The abnormal expression of Ki-67 (20%) revealed the potential proliferation activity of the tumor cells which indicated poor prognosis and high incidence of recurrence. Both the lesions showed the amplification of epidermal growth factor receptor (EGFR), which indicated the high aggressiveness. Pathological results were compatible with a diagnosis of multifocal glioblastoma. We also performed the whole genome sequencing (WGS) of the four lesions. The WGS results revealed that four samples had TERT promoter mutation C228T (chr5:1,295,228:C>T), but none of IDH1/2 mutation, ATRX mutation, and EGFRvIII were detected.

**Fig. 2:**
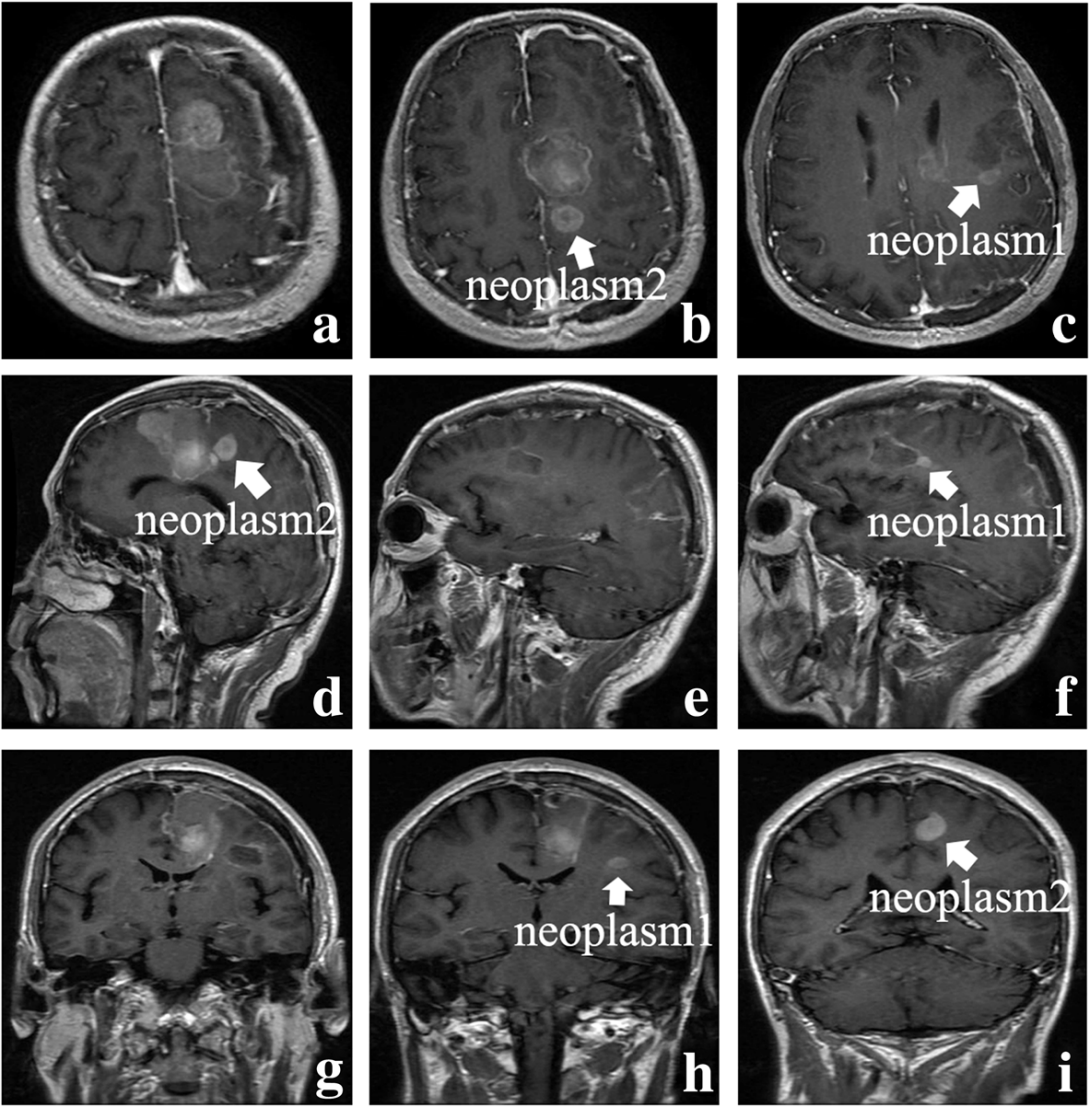
The axial, sagittal, and coronal contrast-enhanced MRI images after 5 weeks of surgery (**a**–**i**). Two new lesions occurred adjacent to the primary lesion area (arrows represent the neoplasms)

The patient presented with no other abnormal neurological signs and his right limb’s strength had improved gradually after operation. There was no recurrence of his epilepsy at this duration. After 5 weeks, a follow-up MRI was performed before radiotherapy and revealed that local tumor control was achieved and the initial resection cavities were seen; however, two new lesions occurred adjacent to the primary lesion areas and exhibited intense enhancement; nevertheless, remarkable mass effect and middle line shift were not observed either (Fig. 2**a**–**i**). Concurrent radiotherapy and chemotherapy were then administered to the patient.

Intensity-modulated radiotherapy (IMRT) targeted these lesions and areas of enhancement. A dosage of 60.0 Gy in 30 fractions were performed and constantly orally administered temozolomide (TMZ), with a dosage of 120 mg (75 mg/m2/day) per day for 46 days were also performed to achieve the concurrent radio-chemotherapy. Side-effects and toxicity such as liver and hematopoietic dysfunction did not occur.

**Fig. 3:**
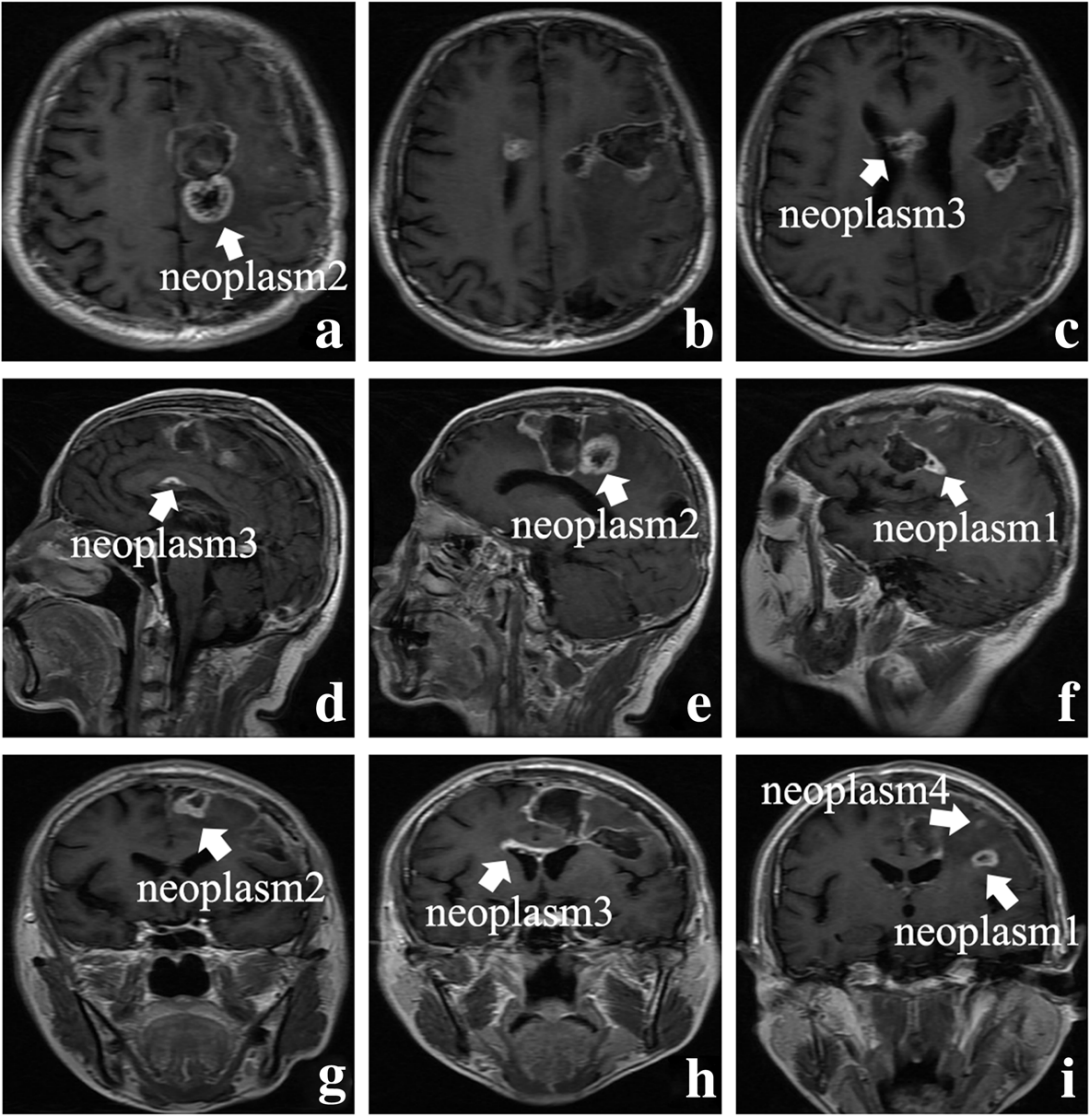
The axial, sagittal, and coronal contrast-enhanced MRI images after 2 cycles of chemotherapy (**a**–**i**). Former two new lesions enlarged distinctly; two more new lesions had emerged again (arrows represent the neoplasms)

The following adjuvant chemotherapy of temozolomide with 240 mg (150 mg/m2/day) for 5 days every 28 days was then administered to the patient. Hematologic and gastrointestinal toxicity was examined every cycle and no significant abnormality was detected. We also monitored the functions of liver and kidney constantly. Dysfunctions of the organs and anaphylaxis were also not seen. After 2 cycles, the repeat MRI revealed that the former two new lesions enlarged distinctly, manifesting ring-enhanced character, and two more new lesions had emerged again, infiltrating the white matter tract of ventricles and parenchyma (Fig. 3**a**–**i**). Eventually, the patient abandoned the chemotherapy after the 2 cycles due to his low quality of life. The patient died 1 year later.

### Case 2

A 71-year-old male presented with a history of headache, left limb weakness, and numbness for 1 month. He also described that discontinuous convulsion of the limbs once occurred when he asked for medical service at this duration. Syndrome of vomiting, hypopsia, and cognitive impairment were not detected.

**Fig. 4.**
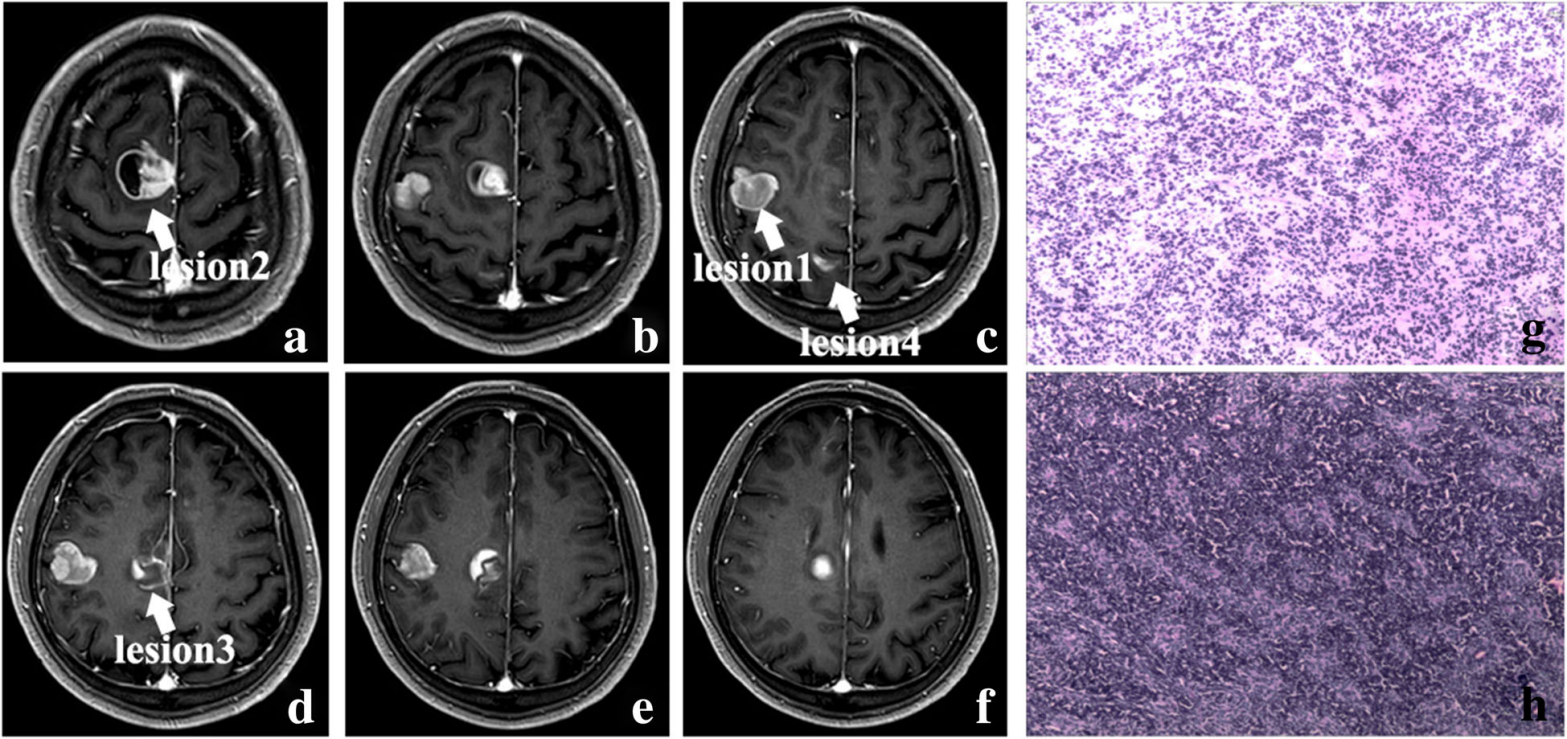
The axial contrast-enhanced MRI images (**a**–**f**) (arrows represent the lesions). The hematoxylin-eosin (HE) staining of intraoperative frozen histologic section and paraffin section (**g**, **h**, × 100)

Gadolinium-enhanced MRI was performed, demonstrating 4 well-defined, highly enhanced lesions located in the right frontal and parietal lobes which were suspicious for metastatic lesions, and one of them was adjacent to the right lateral ventricle (Fig. 4**a**–**f**). PET-CT was also performed and showed hypometabolic foci of FDG corresponding to enhancing lesions seen on the MRI, but the chest, abdomen, and pelvis were negative for any primary lesion.

**Fig. 5:**
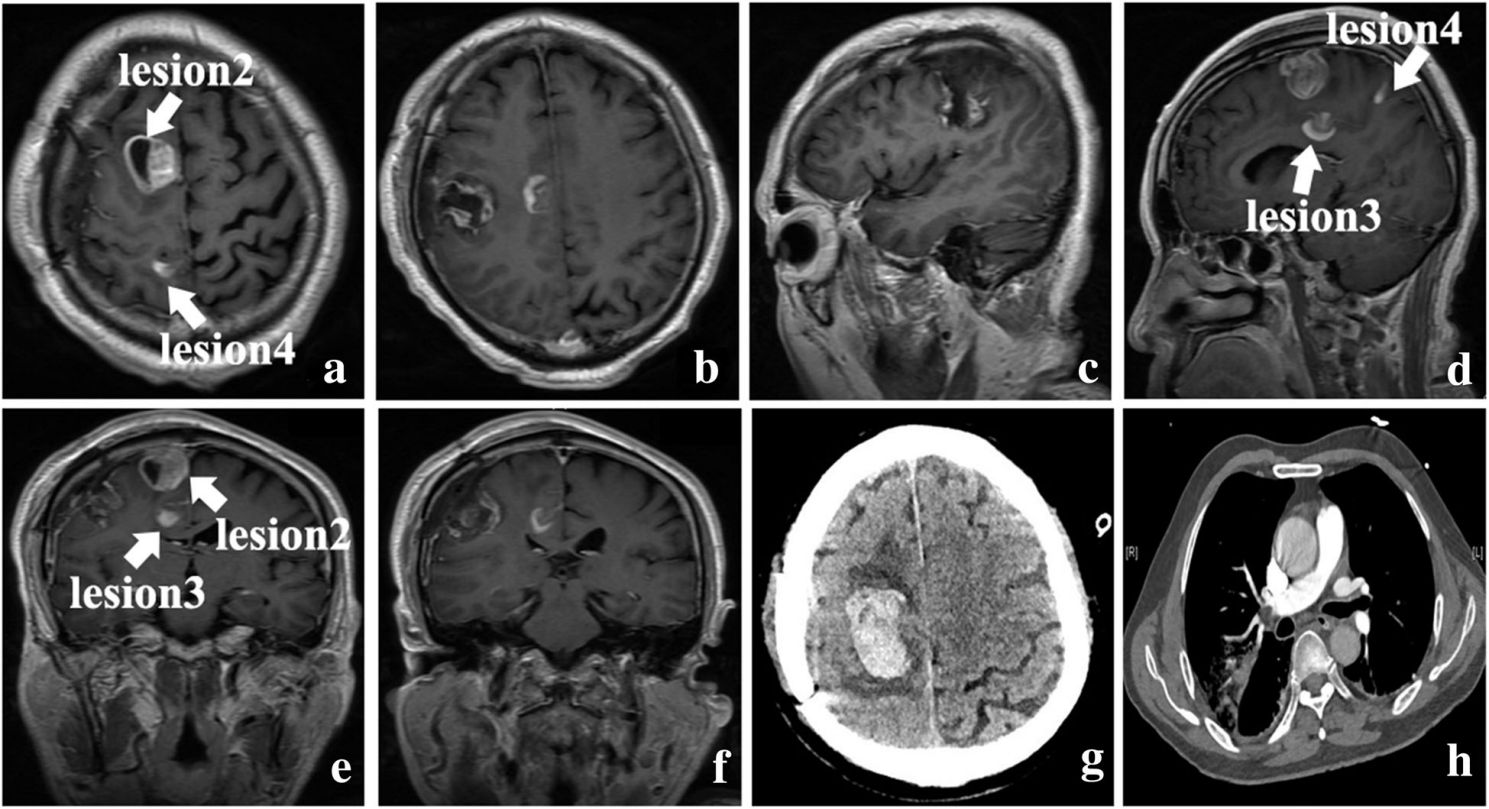
The axial, sagittal, and coronal contrast-enhanced MRI images after surgery (**a**–**f**). Emergent CT scan showed hemorrhage of the residual tumor. **g** The CT angiography of pulmonary artery showed a sign of pulmonary artery embolism (**h**)

To achieve the goals of safe resection and obtain the mass for histopathologic diagnosis, the patient underwent a right frontoparietal craniotomy of the main lesion near the surface of parenchyma with the guidance of intraoperative navigation and intravenous fluorescein. The mass was seen with grey and white appearance, obscure boundary, and moderate blood supply. The gross total resection of the mass around its approximate boundary was also achieved (Fig. 5**a**–**f**) and no significant postoperative abnormal neurological signs were detected.

Histopathological examination revealed infiltrating highly proliferative cells with nuclear atypia which fulfilled the criteria of malignant glioma (Fig. 4**g**, **h**). Immunohistochemical staining demonstrated the highly expressed Ki-67 index (30%), positive GFAP, Olig-2, CD34, CD99, P53, and ATRX whereas, negative IDH1, NeuN, and Vimentin. The final results were also compatible with a diagnosis of multifocal glioblastoma.

The patient suddenly presented a sign of dyspnea at the 10th day after operation, followed with decreased conscious level and blood-oxygen saturation. The emergent CT showed hemorrhage of the residual tumor located in the right parietal lobe closed to the mid-line (Fig. 5**g**); meanwhile, the CT angiography of pulmonary artery showed a sign of pulmonary artery embolism (Fig. 5**h**). Conservative treatment was implemented in consideration of the condition of the patient; eventually, the patient’s family decided not to pursue any further treatment and opted for hospice care and the patient passed away within 11 days of surgery.

## Discussion

Newly diagnosed multiple glioblastoma can be termed as multifocal or multicentric [8]; generally, multifocality is diagnosed when connections between lesions present and tumors always disseminate via parenchymal routes [9]. Previous studies reported that incidence of multifocal glioblastoma is ranging from 0.5 to 20% [10, 11], and patients of multifocal glioblastoma demonstrated worse prognosis. Patil et al. reported that a significantly shorter median overall survival of 6 months was observed in multifocal glioblastoma compared with that of solitary glioblastoma, of which was 11 months, and 2-year survival rates of multifocal glioblastoma were significantly lower than that of solitary glioblastoma [12]. Lasocki et al. reported that patients of multifocal glioblastoma generally live a median survival of 6–8 months from diagnosis [13]. The hypothesized analysis of this dilemma may include higher burden of disease, genetically more aggressive phenotype and inability for gross total resection of the multiple lesions [6]. In our case 1, although the patient received gross total resection of the multiple lesions and additional radio-chemotherapy, new lesions emerged and enlarged shortly after and the patient died 1 year later, demonstrating their strong aggressiveness.

Multiple intracranial lesion imaging characters represent a diagnostic dilemma in that multiple ring-enhancing lesions usually were diagnosed as metastatic entities or brain abcesses [14, 15]. In our cases, contrast enhancement MRI played a role in detecting and assessing these lesions, contributing to the surgical resection safely, and PET-CT was advantageous to discern the primary malignancies in other tissues of body accurately and safely by providing detailed functional and metabolic molecular information. We hold the opinion that it is inevitable to perform the PET-CT to distinguish these lesions under this circumstance; besides, infectious and inflammatory indicators should be taken into consideration to exclude other diseases. Multiple lesions bring great difficulties and risks to surgeons so they are always faced with tough choices. It was uncommon to achieve resection of multiple lesions and 70% of patients underwent resection of only one lesion [9]; thus, tumors tend to invade and relapse sequentially. Recent studies recommend resection of multiple lesions synchronously. Hassaneen et al. reported that aggressive resection of all lesions resulted in a survival duration comparable with that of patients undergoing resection for a single lesion, without an associated increase in postoperative morbidity [12]; thus, the resection of multiple lesions may be a beneficial prognostic factor in these patients. In our case 1, we successfully achieved the resection of all lesions, reducing the tumor burden as much as possible; though higher risks are posed potentially, postoperative complications did not occur either. In our case 2, the patient underwent the resection of a single lesion located in the right parietal lobe; nevertheless, hemorrhage of residual tumor occurred postoperatively. Early detection and treatment of this complication can contribute to improve the clinical outcomes; even removal of intracranial hematoma surgically will be beneficial if necessary. Regrettably, the pulmonary embolism resulting from coagulopathy occurred and made the treatment contradictorily; eventually, the patient died without a chance of re-operation and additional therapy.

Local irradiation therapies like IMRT and three dimensional conformal RT (3DCRT), which cover the gross target volume locally, now remain the standard adjuvant treatments of unifocal glioblastoma due to their lower toxicity and accuracy. In our case 1, we adopted IMRT with a dosage of 60.0 Gy in 30 fractions, and side-effects were not detected during the follow-up period. Recent studies also investigated the safety and efficacy of whole-brain radiotherapy (WBRT) in GBM patients. Lahmi et al. reported their retrospectively study of patients received the WBRT with TMZ and confirmed the safety and efficacy of WBRT, but larger prospective studies are needed to support their results [16]. The Stupp protocol of chemotherapy is widely recommended for newly diagnosed GBM [17]. Liu et al. reported that long-term temozolomide (TMZ) might be an optimal choice for patient with multifocal glioblastoma [7]. In our case 1, the patient received 2 cycles of sequential TMZ chemotherapy after the concurrent radio-chemotherapy, although the general condition of patient remained stable, new lesions emerged, and this might result from the more aggressive phenotype of this disease.

Underlying genetic characteristics of multifocal glioblastoma have not been elucidated clearly. Liu et al. found that multifocal glioblastomas had no IDH1, ATRX, or PDGFRA mutations compared with solitary glioblastomas and were significantly associated with the mesenchymal subtype [8]; Abou-El-Ardat et al. investigated its comprehensive molecular characterization and they found the high frequency of alterations in the 3 GBM core pathways: RTK/PI3K, p53, and RB regulatory pathways, which resemble primary GBMs, suggesting that multifocal glioblastomas develop through parallel genetic evolution [5]. Besides, discordance of IDH mutational status between lesions was also founded in multifocal glioblastoma, indicating the higher heterogeneity compared with unifocal GBM [18]. In our study, although genetic analysis of lesions revealed the distinctive genetic and molecular characteristics of multifocal glioblastoma, more specimens from different patients are needed for evaluating and elucidating their heterogeneity in the future.

Currently, there are still no uniform treatment guidelines for multifocal glioblastoma; thus, further investigation and evaluation are required for unveiling this rare malignancy.

## Conclusions

In summary, we have reported two cases of typical multifocal glioblastoma. The resection of multiple lesions and canonical radio-chemotherapy probably bring survival benefits; meanwhile, further investigation is required for understanding the behavior, management, and outcome of this rare malignancy.

## Data Availability

Not applicable.

## Abbreviations

GBM: Glioblastoma multiforme
CT: Computed tomography
PET-CT: Positron emission tomography-computed tomography
MRI: Magnetic resonance imaging
3DCRT: Three dimensional conformal RT
WBRT: Whole-brain radiotherapy
IMRT: Intensity-modulated radiotherapy
TMZ: Temozolomide
